# Impact of annual hospital volume of high dose interleukin-2 infusions on in-patient mortality in patients with melanoma and renal cell carcinoma

**DOI:** 10.1186/2051-1426-3-S2-P234

**Published:** 2015-11-04

**Authors:** Kathan Mehta, Leonard Appleman, Hong Wang, Ahmad Tarhini, Rahul Parikh

**Affiliations:** 1 University of Pittsburgh Medical Center, Pittsburgh, PA, USA; 2 Department of Medicine, University of Pittsburgh Medical Center Cancer Pavilion, Pittsburgh, PA, USA; 3 University of Pittsburgh, Pittsburgh, PA, USA; 4 University of Pittsburgh School of Medicine, Pittsburgh, PA, USA

## Background

The 2014 expert consensus on high dose interleukin-2 (HD-IL-2) states that, “Treating a minimum number of patients per year is important, as quality depends upon familiarity and repetition” [1]. The minimum annual volume of HD-IL-2 associated with relatively worse outcomes is not known.

## Methods

We analyzed the National Inpatient Sample (NIS), one of the largest publicly-available in-patient dataset in United States (U.S.), which represents a 20% stratified random sample of discharges from all hospitals. The NIS is drawn from all States participating in Healthcare Cost and Utilization Project, and thus represents 95 percent of the U.S. population. Patients with melanoma and renal cell carcinoma (RCC) were identified by using the ICD9 diagnostic codes. From this sample, patients receiving HD-IL-2 were identified by ICD9 procedure code 00.15. Annual hospital volume was calculated using a unique hospital number, available in the dataset. Using Joinpoint regression analysis, which detects change in trend of in-patient mortality with change in annual hospital volume, the hospitals were classified in 3 volume categories (low, medium and high). Multivariate logistic regression was used to identify predictors of in-patient mortality controlling for confounders including age, sex, Charlson comorbidity index, RCC, calendar year, urban location and teaching status of hospital.

## Results

**Figure 1:**
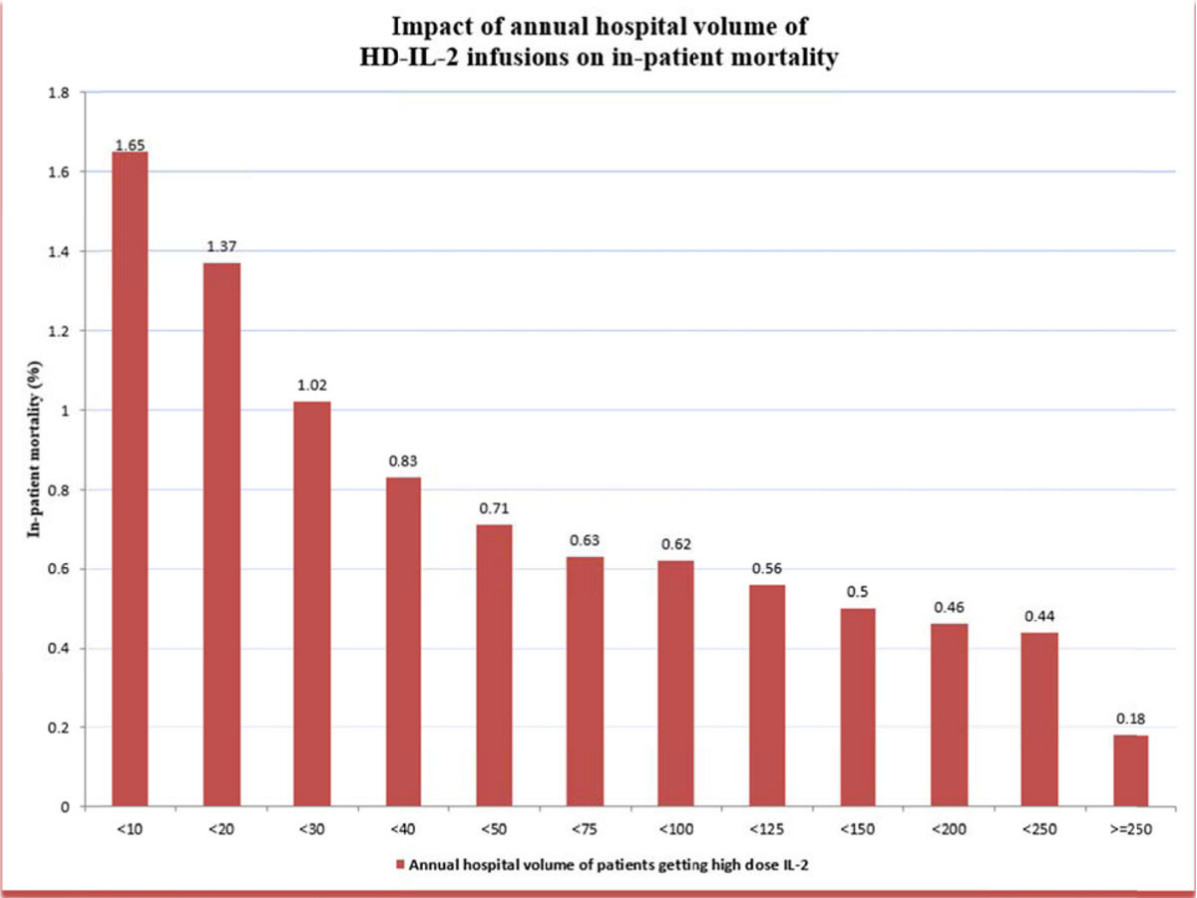


From 2003 to 2011, 29,532 patients (weighted number of patients) with RCC or melanoma who received HD-IL-2 were identified, and 124 died during hospitalization, while receiving HD-IL-2 (0.4%). Higher hospital volume was associated with lower in-patient mortality (Figure 1). The Joinpoint regression identified 3 categories of hospital by annual hospital volume (low: 1-40 per year, medium: 41-120 per year, high >120 per year), which had significant difference in in-patient mortality (0.83%, 0.29% and 0.13% respectively, p=0.0003). On multivariate analysis, the low volume hospitals were associated with higher odds of in-patient mortality (OR 6.1, 95% CI 1.6-23.2, p=0.003) as compared to high volume hospitals. Additionally, hospitals with annual volume of 1-20 per year had even higher rates of inpatient mortality (1.31% vs. 0.13%, p < 0.0001) and multivariate odds (OR 8.9, 95% CI 2.4-33.2, p=0.0006) as compared to high volume hospitals.

## Conclusion

Lower annual hospital volume of HD-IL-2 is associated with higher HD-IL2 related in-patient mortality. Annual hospital volume of less than 20 treatments is associated with 9 time higher risk of in-patient mortality as compared to high volume hospitals.

